# MicroRNAs: emerging driver of cancer perineural invasion

**DOI:** 10.1186/s13578-021-00630-4

**Published:** 2021-06-29

**Authors:** Mei Zhang, Hong-chun Xian, Li Dai, Ya-ling Tang, Xin-hua Liang

**Affiliations:** 1 State Key Laboratory of Oral Diseases & National Clinical Research Center for Oral Diseases & Department of Oral and Maxillofacial Surgery, West China Hospital of Stomatology (Sichuan University), No.14, Sec. 3, Renminnan Road, Chengdu, 610041 China; 2 State Key Laboratory of Oral Diseases & National Clinical Research Center for Oral Diseases & Department of Oral Pathology, West China Hospital of Stomatology (Sichuan University), No.14, Sec. 3, Renminnan Road, Chengdu, 610041 China

**Keywords:** Perineural invasion, Malignant tumors, MicroRNA, Biomarkers

## Abstract

The perineural invasion (PNI), which refers to tumor cells encroaching on nerve, is a clinical feature frequently occurred in various malignant tumors, and responsible for postoperative recurrence, metastasis and decreased survival. The pathogenesis of PNI switches from ‘low-resistance channel’ hypothesis to ‘mutual attraction’ theory between peripheral nerves and tumor cells in perineural niche. Among various molecules in perineural niche, microRNA (miRNA) as an emerging modulator of PNI through generating RNA-induced silencing complex (RISC) to orchestrate oncogene and anti-oncogene has aroused a wide attention. This article systematically reviewed the role of microRNA in PNI, promising to identify new biomarkers and offer cancer therapeutic targets.

## Background

Perineural invasion (PNI) was defined as the presence of tumor cells along the sides of nerves and/or inside the epineurial, perineurial and endoneurial spaces of the neuronal sheath [1, 2], which was first proposed by Drapiewski et al. more than a century ago and was regarded as the fifth route of cancer spread in addition to four well-known ways: direct invasion to surrounding tissues, lymphatic metastasis, hematogenous metastasis and seeding along body cavities [1]. PNI can occur in many malignancies, including head and neck cancer, pancreatic ductal adenocarcinoma, gastric carcinoma, colorectal cancer, breast cancer, cervical cancer, prostate cancer, melanoma and so on [3–5]. The occurrence of PNI in tumors not only brings about pain and dysfunction of involved organs, but it makes curative resection within safe margins difficult and residual tumor cells in or around nerves may favor local postoperative recurrence, infiltration and metastasis, which is identified as a vital contributor to the poor clinical prognosis [6–8]. So, tremendous amount of research was launched to comprehend pathogenesis of PNI and the cognition to PNI was deepening.

**Fig. 1:**
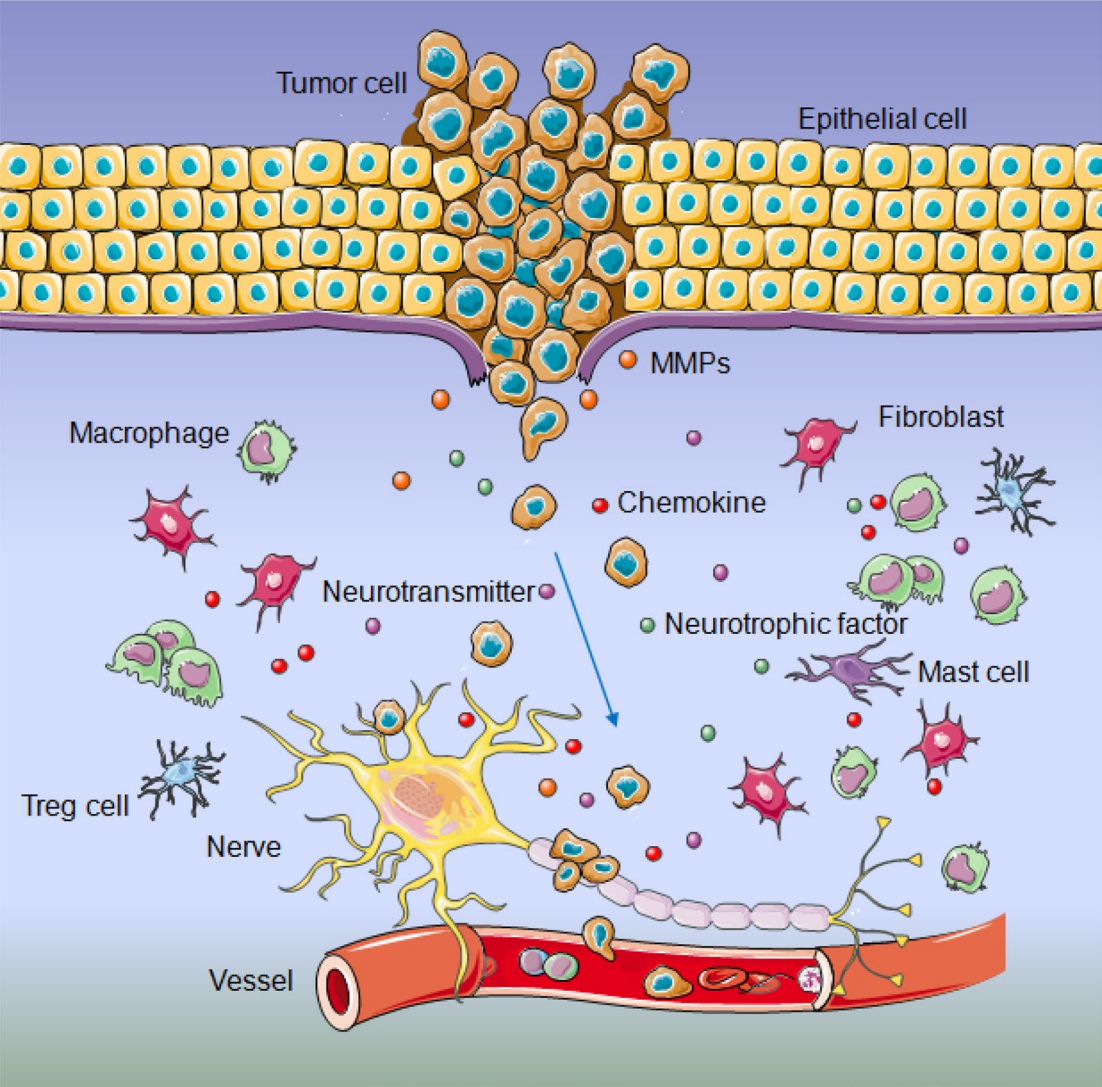
Schematic representation of perineural niche facilitating PNI. Various molecules (neurotrophic factors, neurotransmitters, chemokine, MMPs) and cells (fibroblasts, macrophages, Treg cells, mast cells) in the nerve-tumor niche impelled tumor cell displayed a tendency to encroach on nerves

The initial hypothesis of PNI is the ‘low-resistance channel’ that tumor cells spread passively along the connective tissues covered the nerves, or through the small nerve branches and the perforating vessels of the nerve beams, which is presented relying on the direct observation of the ultrastructure due to technical restrictions [9, 10]. Recently, the development of molecular biology techniques and new invasion models has deepened the perception of PNI, whose formation is not a consequence of single factor, but a continuous, elaborate and active ‘mutual attraction’ multistep process among malignant cells, peripheral nerves and stromal cells in perineural niche. Once tumor cells invaded toward nerve, they created tumor microenvironment. Secreted proteins from tumor encouraged neurite outgrowth, remodeling and axonogenesis directing at the neoplastic front, which in turn initiated a bi-directional communication between tumor cells and nerve [7]. During this process, nerve injury and repair were involved, which making them more vulnerable to tumor invasion [7, 11]. Dying nerve secreted a series of neurotransmitters (catecholamines, acetylcholine and neuropeptides), membrane-anchored proteins (MUC1 and L1CAM), matrix metalloproteinases (MMPs), non-coding RNAs (miRNA, lncRNA and cirRNA), neurotrophic factors (GDNF, NGF, BDNF, NT3 and CSF1) and chemokines (CXCL12, CCL2, CCL5 and CX3CL1), which could be released in niche or specifically bind with their receptors (GFRα1, TRKA, TRKA, TRKC, CSF1R, CXCR4, CCR2, CCR5 and CX3CR1) expressed in tumor cell, thus favoring both the inflammatory response in niche and the tendentious movement of the tumor toward the nerve. Further, tumor-associated inflammation recruited stromal cells, such as fibroblasts, macrophages, regulatory T cells (Treg cells) and mast cells into the perineural niche, which established a bridge during the crosstalk between tumor cells and nerve [12, 13] (Fig. 1). Among these molecules, the role of miRNA in the PNI of malignance was thrown into sharp focus recently [14, 15]. Herein, we summarized the possible direct or indirect dysregulated miRNAs along with identified molecular cues involved in nerve remodeling and perineural spread, reviewed crosstalk between miRNAs and other PNI related molecules as well as stromal cells to guide target treatment decision-making regarding using the new miRNA biomarker in cancer patients.

## Biogenesis of miRNAs

MiRNAs are a class of single-stranded non-coding RNAs with a length of 18–25 nucleotides. The biosynthesis of miRNAs is partitioned into canonical and non-canonical pathways [16, 17]. As noted in Fig. 1, the canonical pathway of miRNAs formation starts with transcription, during which primary miRNA precursors (pri-miRNAs) are generated by RNA polymerase II (Pol II) and III [18]. Microprocessor complex composed of Drosha (RNA-specific endoribonuclease III) and DGCR8 (double stranded RNA binding protein) excises the hairpin structure in the pri-miRNAs, and a 70-nucleotide stem loop known as precursor miRNA (pre-miRNAs) is produced in the nucleus [19]. Then, nuclear export protein exportin 5 (Exp5) forming complex with the GTP-binding nuclear protein Ran (Ran-GTP) exports pre-miRNAs to the cytoplasm, where the pre-miRNA is cleaved by Dicer, a helicase-RNase III hybrid, generating a mature double-stranded miRNA duplex [20, 21]. Subsequently, the guide strand of the miRNA duplex is incorporated onto argonaute (Ago) protein complex forming RNA-induced silencing complex (RISC), and the passenger strand is deemed non-functional and degraded once released [22].

**Fig. 2:**
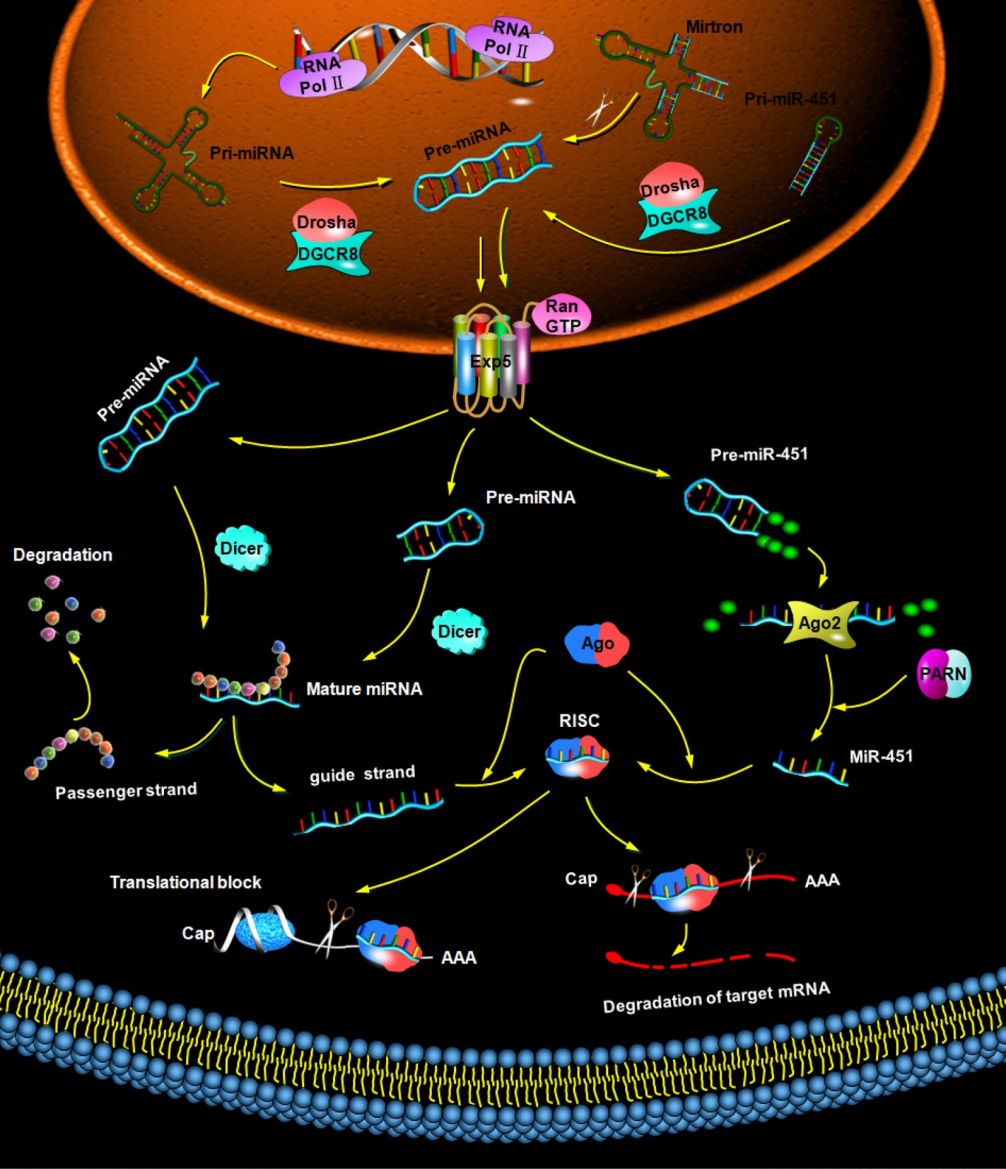
Biogenesis of miRNAs. Canonical microRNA biogenesis, miRNAs formation starts with transcription, during which pri-miRNAs are generated by RNA Pol II and III. Microprocessor complex composed of Drosha and DGCR8 excises pri-miRNAs, and pre-miRNAs is produced in the nucleus. Then, the nuclear Exp5 forming complex with the Ran-GTP exports pre-miRNAs to the cytoplasm, where the pre-miRNA is cleaved by Dicer, generating a mature double-stranded miRNA duplex. Subsequently, the guide strand of the miRNA duplex is incorporated onto Ago protein complex, forming RISC to recognize and interact with the 3’-UTR of their targets. Conversely, the passenger strand is deemed non-functional and degraded once released. Drosha-independent miRNAs biogenesis, the Drosha-independent mirtron genes do not undergo Drosha cleavage but demand mRNA splicing and debranching forming a stem-loop structure analogous to the pre-miRNA synthesized in the canonical approach. The subsequent generation process of miRNAs is similar to canonical pathway. Dicer-independent miRNAs biogenesis, the Dicer-independent pathway is mainly involved in the generation of miR-451 family, which is processed by the microprocessor complex Drosha/DGCR8 in the nucleus. Then, a short pre-miRNA was generated and transported to the cytoplasm by Exp5. The pre-miR-451 is cleaved by Ago2 and trimmed by PARN engendering the mature miR-451, which integrated into Ago to constitute the functional core of RISC

The non-canonical pathways of miRNA biogenesis include Drosha-independent and Dicer-independent pathways [23, 24] (Fig. 2). Drosha-independent mirtron genes do not undergo Drosha cleavage but demand mRNA splicing and lariat debranching (intron gene), forming a stem-loop structure analogous to the pre-miRNA synthesized in the canonical approach [25, 26]. The Dicer-independent pathway, another non-canonical miRNA biogenesis approach, is mainly involved in the generation of miR-451 family, which is processed by the microprocessor complex (Drosha/DGCR8) in the nucleus. Then, a short pre-miRNA was generated and transported to the cytoplasm by Exp5. The pre-miR-451 is cleaved by Ago2 and trimmed by PARN, engendering the mature miR-451, which integrates into Ago to constitute the functional core of RISC [25].

After the formation of RISC complex, ‘the seed’ domain at the 5ʹ end could recognize and interact with the 3ʹ-untranslated region (3ʹ-UTR) of its target gene through complementary base binding [27, 28], inducing mRNAs degradation or translational repression of target mRNAs, leading to ‘gene silence’ and orchestrating broad physiological processes, including cell proliferation, differentiation, apoptosis (programed cell death), cell cycle, tissue development, energy biosynthesis, cellular metabolism and pathological process, such as cardiovascular disorders, neurological disease, endometriosis and diabetes [29–31].

Besides, a tremendous amount of document reported that miRNAs were also involved in the malignant transformation and tumor progression by exerting pivotal effects on oncogenes and suppressor genes through a series of signaling axis including NF-κB, MAPK, PI3K/AKT/mTOR and Wnt/β-catenin [32, 33].

## MiRNA alterations associated with PNI

### MiRNA promoting PNI

#### MiR-21

MiR-21, encoded by the MIR21 gene located on 17q23.2, is one of the most studied miRNAs in various fields, including growth and development, stem cell biology, aging and oncology [34]. It can also participate in nerve remodeling through orchestrating growth signaling, accelerating axon growth [35] and inhibiting neuron death [36]. Sakai et al. confirmed that miR-21 was consistently upregulated after peripheral nerve injury in the dorsal root ganglion (DRG) and was related to neuropathic pain [37]. Elevated miR-21 expression was regulated by IL-6 in the DRG following partial sciatic nerve ligation [38]. In addition, miR-21 was reported to be highly expressed in nerve-derived tumors, including glioblastoma, malignant peripheral nerve sheath tumor, vestibular schwannomas, glioblastoma multiforme [39–41]. In 2012, Teplyuk et al. certified that compared with tumors in remission and nonneoplastic conditions, the level of miR-21 was significantly increased in the cerebrospinal fluid of lung and breast cancer patients with brain metastasis using microRNA profiles [42]. Later, Singh et al. used patient-derived stem cell lines from lung-to-brain metastases to investigate the modulatory effect of STAT3 in brain metastasis initiating cells and found STAT3 and miR-21 functioned as cooperative orchestrators of stemness and tumor initiation in lung-derived brain metastases [43]. Loredana et al. demonstrated that miR-21, secreted by melanoma cells in small extracellular vesicles contributed to brain metastasis of melanoma patients [44]. These suggested that tumor cells expressing miR-21 had a disposition to migrate towards brain tissue.

Further, increasing evidence showed that miR-21 contributed to the PNI of certain non-neural origin tumors. Yu and coworkers demonstrated that miR-21 might promote PNI of oral carcinoma through inhibiting PTEN [15]. Robyn et al. authenticated that miR-21 was higher expressed in PNI group than in non-PNI group of prostate cancer [45]. Adam et al. identified that miR-21 combining with miR-23a and miR-27a posed as cooperative repressor of a network of tumor-suppressor genes including PDCD4, B-cell translocation gene 2 (BTG2) and neural precursor cell expressed developmentally downregulated 4-like (NEDD4L) in PNI of pancreatic cancer. Inhibition of miR-21 could reduce cell proliferation of pancreatic ductal adenocarcinoma (PDAC) in vitro and the growth of xenograft tumor in vivo and synergistic inhibition effects could be observed via simultaneously silencing miR-23a and miR-27a [46]. In cholangiocarcinoma, miR-21 could potentially inhibit RECK expression, thus facilitating PNI [47]. This indicated that miR-21 potentially contributing to PNI, however, the detailed mechanisms of miR-21 on PNI, such as alteration in specific gene expression levels or domination the accessibility of signaling, has still not been characterized.

#### MiR-99 family: miR-99 and miR-100

MiR-99 family, whose origin can be traced back to the bilaterian ancestor, is universally known as one of the oldest miRNA families [48]. Current studies have found three members of the miR-99 family: miR-99a, miR-99b, miR-100 [49]. Robyn et al. discovered that the level of miR-99b and miR-100 were higher in PNI group than in non-neurotropic prostate cancer using microRNA microarray and Affymetrix Genechips, moreover, concurring with above data, neurotropic prostate cancer cells were inclined to exhibit a lower metallothionein level relevant to non-invasive tumor cells [45]. Additionally, macrophages can be classified into M1 and M2 phenotypes. M1 polarized macrophages possess anti-tumor functions whereas M2 tumor associated macrophages (TAMs) promote tumor growth [50]. The level of miR-99a and miR-99b are found to be enriched in M2 macrophages compared to unstimulated macrophages [51]. Jaiswal et al. demonstrated that miR-99a overexpression could downregulate the expression of M1 macrophages markers [52]. And the expression of exosome-derived miR-99a was proved to be upregulated in M2 macrophages [53]. Wang et al. also reported that miR-100 promoted the markers expression of M2-associated phenotypes in macrophages [54]. Immunohistochemical analysis revealed that endoneurial macrophages are abundant in nerves invaded by cancer compared with normal nerves. Nerve derived macrophages, recruited and activated by cancer cells in neuro-tumor microenvironment have been demonstrated to assist tumor cells in invading towards nerve through secreting GDNF in vitro [55]. Together these studies indicated that association between M2 macrophages and miR-99 family might be a potential mechanism for modulating PNI and the exact mechanism awaited further investigation.

#### MiR-17

MiR-17 belongs to the highly conserved polycistronic miR-17–92 cluster, which locates in chromosome 13 open reading frame 25, encoding 6 mature miRNA molecules (namely, miR-17, miR-18a, miR-19a, miR-19b, miR-20a and miR-92a) [56]. MiR-17 contributed to the onset of multiple sclerosis, a neuro-destructive autoimmune disease through augmenting TH17 responses elicited by the activation of PI3K–AKT–mTOR axis [57]. In a spinal cord injury (SCI) model, overexpression of miR-17 was reported to facilitate glial scar formation and suppress the neurofilaments regeneration through targeting PTEN and stimulating the PI3K/Akt/mTOR pathway [58]. In primary sensory neurons, inhibition of miR-17-5p by PEITC could promote neurite growth [59]. MiR-17 may be associated with the increased lumbar radicular pain after disc herniation, possibly via a TNF-driven pro-inflammatory mechanism [60]. Downregulated miR-17-5p involved in nerve cell damage [61]. Gene Expression Omnibus (GEO) data set of microarray reveled that the expression level of miR-17-5p in patients with brain metastasis was significantly upregulated than that in situ carcinoma, indicating that it might participated in the brain metastatic process of breast cancer [62]. A recent study validated that miR-17 stimulated by GFRα2 might exert a promoting PNI effect by downregulating tumor suppressor gene PTEN in pancreatic cancer, which provided new insights for future research into the role of miR-17 in PNI [63].

#### MiR-23a/24–2/27a family

The miR-23a/24–2/27a cluster, an intergenic miRNA cluster, is located at chromosome 19p13.12 in the vertebrate genome and encodes an about 2159nt pri-miRNA transcript embodying three functional miRNAs (namely, miR-23a, miR-24 and miR-27a) [64]. The members of this cluster are involved in cell differentiation and cycle control [65]. In peripheral nerve injury, miR-24-3p could be squeezed by TNXA-PS1 to upregulate the expression of specificity phosphatase 1 (Dusp1), thus accelerating migration of schwann cells, a key component in neural repair and regeneration and orchestrating the cues for PNI of tumor [7, 66]. MiR-24 suppressed oligodendrocyte precursor cell differentiation, which allocated nutrients to neurons in spinal injury through upregulating the level of PDGFRa, NG2, IL-6 and TNF-α and downregulating the expression of MBP and ADM [67]. Similarly, Deng et al. noted that miR-24 inhibitor activated the expression of HMOX1, thereby decreasing oxidation and inflammatory response as well as improving neurological functional deficits in the rats with cerebral vasospasm after subarachnoid hemorrhage [68]. In primary malignant brain tumor glioblastoma, miR-23 was downregulated along with its target ATP5A1 or ATP5B high expression by miRNA microarray [69]. However, miR-24 was demonstrated to favor proliferation, invasion and viability of glioma cells by targeting ST7L and activating β-catenin/Tcf-4 signaling axis [70, 71]. An integrated statistic analysis authenticated that miR-23a collaborating with miR-27a could promote PNI in PDAC [46]. In prostate cancer, higher expression of miR-24-2 and miR-27a was validated to experience a transformation from non-PNI prostate tumor to a tumor with PNI [45]. Thus, miR-23a/24-2/27a cluster could use as a potential diagnostic signature for PNI of tumor patients.

#### MiR-15 family

Similar to above pro-PNI miRNAs is the miR-15 family, which contains six members (miR-15a, miR-15b, miR-16-1, miR-16-2, miR-195, miR-497) and are encoded by intron region of the DLEU2 transcript positioned in the antisense orientation of the 13q14.3 locus [72]. Recent discoveries confirmed ectopic expression of miR-15 family by cancer cells correlates with nerve invasion. As corroborated in prostate cancer, increased expression of miR-15a-2 and miR-195 was detected, which might be attributable to PNI through inhibiting metallothionein expression [45]. Also, miR-16 overexpression induced upregulation of Bcl-2 and suppression of apoptosis, and miR-16 inhibitor enhanced the sensitivity of U251MG/TR cells to temozolomide in neurogenic glioma [73]. Promotion of miR-497 concurrent with increased IGF1R and IRS1 conferred glioma cells resistance to temozolomide [74]. Conversely, Yang et al. reported that miR-16 impaired invasiveness of human glioma cell by downregulating the expression of NF-κB1 and MMP9, which were prominent promoters of PNI [75]. Krell et al. found that miR-16-5p mimics greatly increased sensitivity of A172 to temozolomide [76]. MiR-15a/16 and miR-132 could synergistically suppress proliferation, migration and invasion via directly targeting Sox5 in pituitary tumor [77]. Concurrent upregulation of miR-195 and miR-15b could suppress migration and invasion of glioma cell via post-transcriptionally downregulating SALL4 expression [78]. Treatment with miR-195-5p inhibitor could effectively increase YAP1 and TEAD1 expression, thus promoting glioma progression [79]. Upregulation of miR-195 could competing sponge FASN to diminish proliferation, migration and invasion of IOMM-Lee cells in malignant meningioma [80]. In addition, inhibition of miR-15a/16 alleviated neuropathic pain through upregulating the expression of GRK2, and GRK2 silence reversed the protective effects of miR-15a/16 inhibition in neuropathic pain [81]. MiR-195 level was elevated in a rat infraorbital nerve chronic constriction injury model along with decreased Patched1 expression [82]. Loss-of-miR-497 could inhibited OGD-mediated N2A cell death after transient focal cerebral ischemia in mouse brain [83].

#### Other miRNA promoting PNI

**Fig. 3:**
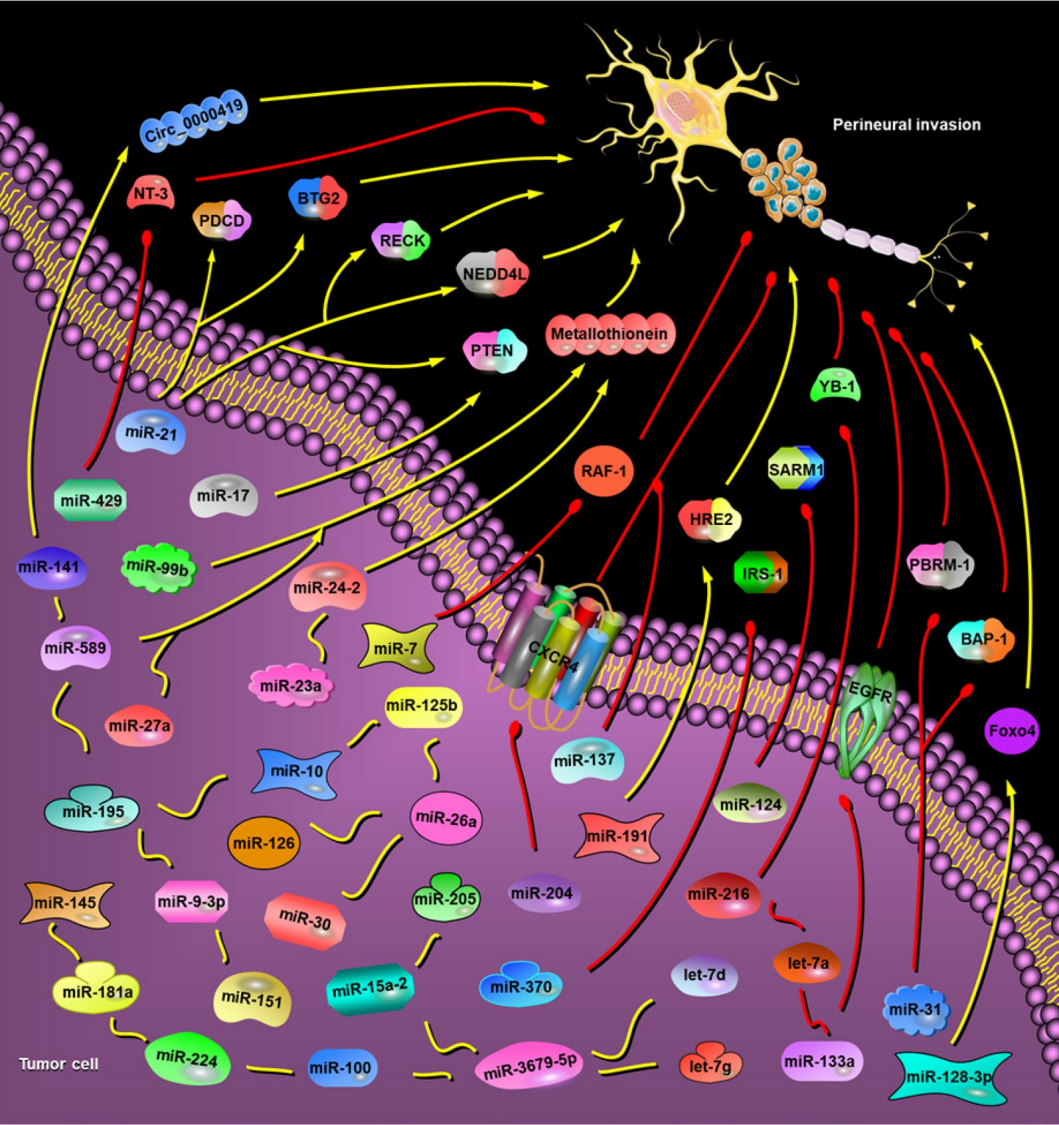
Possible regulatory mechanism of miRNAs in PNI of tumor. Pro-PNI miRNAs: miR-15a-2, miR-195, miR-224, miR-10 (a/b), miR-125b (− 1/2), miR-30a/b/c-2, miR-26a (− 1/2), miR-126, miR-145, miR-151, miR-99b, miR-100, miR-181a-1, miR-191, miR-21, miR-23a, miR-27, miR-17, miR-191, miR-3679-5p, miR-9-3p and miR-205, miR-589-3p, miR-128-3p, miR-24 and miR-27a. Anti-PNI miRNAs: miR-31, miR-370, miR-216, miR-124-3p, miR-133a, miR-204, miR-137 and miR-7. Dual role of miRNAs family in PNI: miR-200 family, miR-141 (pro-PNI), miR-429 (anti-PNI); Let-7 family, let-7a (anti-PNI), let-7d and Let-7g (pro-PNI)

A list of miRNAs related to PNI of prostate cancer was screened out by miRNA microarray, including miR-224, miR-10 (a/b), miR-125b (− 1/2), miR-30a/b/c-2, miR-26a (− 1/2), miR-126, miR-145, miR-151, miR-181a-1 and miR-191 in a recent study [45]. In addition, under the chronic hypoxia condition, the transcription of miR-191 could be initiated by increasing hypoxia-inducible factor-1 (HIF-1) and subsequent bind to HRE2, thus boosting PNI of pancreatic cancer, eventually [84]. High expression of miR-128-3p was proved to be essential for PNI, and miR-128-3p could regulate EMT by directly sponging its downstream target gene FOXO4 to activate TGF-β/SMAD and JAK/STAT3 axis in colorectal cancer [85]. Li et al. verified that miR-3679-5p interacting with lncRNA-CYTOR might enhance PNI through encouraging MACC1 expression in colorectal cancer [86]. In sinonasal squamous cell carcinoma, miR-9-3p was reported to be overexpressed and relevant to PNI [87]. Similarly, miR-205 overexpression was also revealed in cutaneous squamous cell carcinoma and closely associated with PNI [88]. In gastric cancer, miR-589-3p could interact with plasma hsa_circ_0000419 to promote PNI [89] (Fig. 3).

### MiRNA suppressing PNI

#### MiR-31

The miR-31, located in chromosome band 9p21.3, is a momentous peacemaker in fertility, embryonic development, bone formation, myogenesis and immune system function and is characterized as a tumor suppressor [90]. MiR-31 could repress invasiveness and the migratory ability of glioblastoma cells by regulating the level of RGS4, EMP1 and TGFBR1 [91]. It was also validated that the enforced expression of miR-31 significantly potentiated the cytotoxic effects of temozolomide on the glioblastoma cells through the inhibition of STAT3 activation [92]. Significantly downregulation of miR-31 occurred in brain-metastatic carcinomas through examining the miRNA expression of 3 primary colorectal cancers and matched brain-metastatic carcinomas [93]. During the process of nerve remodeling, miR-31 level was significantly elevated at 7 days in three nerve injury models, including ventral root transection, dorsal root transection and spinal nerve ligation [94]. Consistently, Xu et al. reported that miR-31-5p was up-regulated in the serum of rats with spinal nerve ligation surgery at days 3, 7 and 13 post-surgery [68].

A recent research in intrahepatic cholangiocarcinoma noted that PNI tumor with low miR-31 expression showed increased expression of BAP-1 and PBRM-1, indicating that miR-31 might repress PNI through disturbing the expression of BAP-1 and PBRM-1 [95], and delivery of agomir-31 might be an available anti-PNI therapeutic strategy in cholangiocarcinoma.

#### MiR-133

MiR-133, identified as an antioncogene in various types of cancers may also have a role in anti-PNI [96, 97]. In pancreatic cancer, miR-133a could exert the anticarcinogenic activity by directly targeting FSCN1 [98], conversely, inhibition of miR-133a by LncRNA XIST could upregulate EGFR expression to accelerate PNI of pancreatic cancer [99]. Chang et al. found that miR-133a-3p mimic upregulated the level of p-p38 MAPK in peripheral nerves and induced neuropathic pain, and administration of miR-133a-3p inhibitor could alleviate neuropathic pain [100]. Raheja et al. identified that compared with healthy control, miR-133a-3p was upregulated in patients with amyotrophic lateral sclerosis, a debilitating neurodegenerative disorder [101]. Besides, approximately 50% decrease of miR-133a level was detected at 3 and 6 months after sciatic nerve entrapment using RT-PCR analysis [102]. Lu et al. revealed that miR-133b could accelerate neurite extension and axon regeneration through modulating RhoA expression in primary cortical neurons [103]. Besides, miR-133b has been charactered as a specific miRNA of ischemic cerebral, and was closely associated with neurite remodeling and functional recovery [104]. Kim et al. noted that miR-133b was abundantly and specifically expressed in mammalian midbrain dopaminergic neurons, and it played a vital role in the pathogenesis of Parkinson’s disease [105]. Upregulation of miR-133b suppressed NF-κB activation and oxygen–glucose deprivation-caused cell apoptosis, thereby alleviating neuronal injury in cerebral ischemia [106]. Taken together, these findings showed that it was possible that miR-133 could trigger PNI by modulation of neural regeneration and tumor cell invasiveness.

#### MiR-204

MiR-204, which stems from the sixth intron of the transient receptor potential melastatin 3 (TRPM3) and its expression is regulated by the promoter of TRPM3, has been verified to participate in eye development and neural differentiation processes [107]. The data from microarray analysis revealed that miR-204 was highly abundant and contributed to growth and development of the axons in sympathetic neurons [108]. Whereas, another study revealed that overexpression of miR-204 curbed the length and extension of neurites in dorsal root ganglia neuron [109]. In addition, Wang et al. delineated that the level of miR-204 was increased in rats model with optic nerve injury, and it enhanced the apoptosis of retinal cells through upregulating the expression of MyD88, TLR4 and NF-κB and downregulating the level of neuroprotective factor GAP-43 [110]. Inhibitor of miR-204 contributed to the repair of injured nerves by disinhibiting Nrn1 expression and derepressing the pro-apoptotic function of schwann cells [111]. MiR-204 was also involved in age-associated degradation in hippocampal function through negatively regulating EphB2 and NMDAR-dependent LTP [112]. Significant decrease of miR-204 was observed in malignant peripheral nerve sheath tumors [113]. Data from online tools and mechanistic cues revealed that miR-204-dependent MALAT1 restrained PNI through targeting to 3’UTR of CXCR4 in human hilar cholangiocarcinoma, providing the evidence that miR-204 might act as a potential anti-PNI therapeutic strategy in cholangiocarcinoma [114].

#### Other miRNA suppressing PNI

MiR-370, extensively covered as a tumor-suppressor in numerous tumors, has a low expression in oral squamous cell carcinoma tissue with PNI, and it gives scope to carcinogen effect through targeting IRS-1 [115]. MiR-7 was also demonstrated to inhibit PNI of OSCC through targeting RAF-1 and PIK3CD [116]. Helena et al. found decreased miR-137 expression in oropharyngeal cancerous tissues by analyzing the miRNA expression in paired cancerous and normal tissues from 77 HNSCC patients, and lower miR-137 levels correlated with increased incidence of PNI [117]. MiR-124-3p, negatively modulated by lncRNA OGFRP1, was affirmed to impair PNI of prostate cancer through regulating SARM1 level [118]. This was consistent with biological properties of miR-124-3p, which was abnormally expressed in many malignancies and commonly acted as a tumor suppressor. For example, in prostate cancer, miR-124 attenuated growth and invasion through PACE4 pathway [119]. In breast cancer, miR-124 has been authenticated to restrain cell growth and migration via targeting flotillin-1 [120]. In hepatocellular carcinoma, miR-124 orchestrated EMT and cytoskeletal events through suppressing oncogenes ROCK2 and EZH2, ultimately curbing invasion and metastasis [121]. Moreover, miR-216 could target 3'-UTR region of YB-1, implicating in decreased expression of MMPs, and disrupting PNI of pancreatic cancer, eventually [122] (Fig. 3).

### Dual role of miRNA in PNI

#### MiR-200 family

The miR-200 family, consisting of five members (including miR-200a, miR-200b, miR-200c, miR-141 and miR-429) was separated into two clusters, namely miR-200a/200b/429 (located on chromosome 1p36) and miR-141/200c (located on chromosome 12p13) based on the different genomic loci and regarded as hallmarks of epithelial cells [122]. MiR-200 family induced neurite formation while inhibition of miR-200 boosted neuronal proliferation through targeting SOX2 and KLF4 genes [123, 124]. Overexpression of miR-429/200a/200b could attenuate CoCl_2_-induced neuronal apoptosis through blocking Notch1 signaling pathway [125]. Illumina microRNA microarray chip analyses of primary gastric adenocarcinoma and matched brain metastatic adenocarcinoma revealed that miR-200b-3p and miR-141-3p was significantly upregulated in brain metastatic lesions, and the expression of ZEB2, the top ranked target gene for miR-200b-3p and miR-141-3p in online microRNA database was markedly downregulated in some brain metastatic samples, thereby, scholars speculated that expression of miRNA-200 family members were correlated with brain metastases of gastric adenocarcinoma [126]. 4 members of miR-200 family including miR-200a, miR-200b, miR-200c and miR-141 was highly elevated in cerebrospinal fluid samples with brain metastasis, but not in the control or glioblastoma cases, allowing discrimination between primary brain cancer and metastatic brain tumors [42]. The result of bioinformatics analysis by Tao et al. affirmed that miR-141 interacting with circ_0000419 endowed tumor cells with PNI in gastric cancer [89], however, another member of miR-200 family, miR-429 was corroborated to arrest PNI through targeting neurotrophin-3 (NT-3) in pancreatic cancer [14], suggesting the members of miR-200 family had the potential to be novel biomarkers for PNI screening.

#### Let-7 family

Let-7 was first identified in *C. elegans* by Reinhart et al. [127]. Subsequently, let-7 homologs were discovered in varieties of species scoping from vertebrates to mollusks [127]. In humans, 10 mature subtypes of the let-7 family have been described (let-7a, let-7b, let-7c, let-7d, let-7e, let-7f, let-7 g, let-7i, miR-98 and miR-202), in which mature let-7a and let-7f were coded by precursor sequences let-7a-1, let-7a-2, let-7a-3 and let-7f-1, let-7f-2, respectively [128]. Let-7 has been already shown to be important for both neurogenesis and neural regeneration. It could affect the phenotype of schwann cells by directly targeting the nerve growth factors including NGF and BDNF to facilitate the axon growth and function recovery after sciatic nerve injury [129, 130]. Ntn1, one of the most studied axon guidance factors, could build the neural conduction pathways and direct the migration of neuronal cells through binding to its receptor Dcc. Let-7 could control the amount of Ntn1 secretion from schwann cells to regulate nerve regeneration [131]. let-7 levels were elevated in cerebrospinal fluid from patients with Alzheimer’s disease, and in vitro let-7 could be released by dying neurons, where it initiated neurodegeneration through neuronal TLR7 signaling [132]. However, Fernández et al. reported that let-7 blocked the Irs2-driven formation of rosettes through repressing the p53 pathway signaling in the early telencephalic neuroepithelium [133]. Besides, dysregulation of let-7 family was considered to associate with PNI of tumor, and their biological role in PNI is inconsistent. For instant, Bárbara et al. confirmed that downregulation of let-7a was relevant to PNI of oral cavity and oropharynx squamous cell carcinoma [134]. In HNSCC, it was reported by Tomasz et al. that increased let-7d collaborating with decreased miR-205 expression was connected to the presence of PNI [135]. In prostate cancer, let-7 g overexpression was demonstrated to boost PNI [45], however, the functional effect of let-7 family in PNI is not systematic or sufficient, so it deserved to be investigated further.

#### MiR-199 family

The miR-199 family consists of two individual miRNAs, miR-199a and miR-199b, which was first cloned from human osteoblast sarcoma cells and mouse skin tissues by Lagos-Quintana et al. in 2003. Bao et al. illustrated that miR-199a-5p could defend the spinal cord against ischemia/reperfusion-mediated injury through inhibiting ECE1 expression in rats [136]. However, Gao et al. revealed that decreased miR-199a protected against neuronal damage and contributed to functional recovery through upregulation of SIRT1, deacetylation of p53 and the activation of mammalian-target-of-rapamycin signaling pathway in Spinal Cord Injury of rats [137–139]. Inhibition of miR-199a could encourage neuroprotection through upregulating Sirtl expression in the brain ischemic tolerance of rats [140]. Lv et al. demonstrated that miR-199a could be sponged by lncRNA-Map2k4, which subsequently upregulated FGF1 expression and stimulated neuron growth of mouse spinal cord [141]. Studies suggested that abnormal expression of miR-199 also interposed PNI of tumor. In prostate cancer, it was reported that miR-199 was highly expressed in PNI tumors relevant to non-PNI tumors [45], oppositely, low miR-199b was associated with the presence of PNI in head and neck squamous cell carcinoma [142] (Fig. 3) (Table 1).

**Table 1:** Dysregulation of miRNAs in PNI of tumor

Cancer type	miRNA	Regulation	Target	References
Pancreatic cancer	miR-429	Downregulated	NT-3	[14]
	miR-191	Upregulated	HRE2	[45, 84]
	miR-21, miR-23a/miR-27a	Upregulated	PDCD、BTG2、NEDD4L	[46]
	miR-17	Upregulated	PTEN	[63]
	miR-133a	Downregulated	EGFR	[99]
	miR-216	Downregulated	YB-1	[122]
Cholangiocarcinoma	miR-21	Upregulated	RECK	[47]
	miR-31	Downregulated	BAP-1, PBRM-1	[95]
	miR-204	Downregulated	CXCR4	[114]
Gastric cancer	miR-589-3p, miR-141	Upregulated	circ_0000419	[89]
Colorectal cancer	miR-128-3p	Upregulated	Foxo4	[85]
	miR-3679-5p	Upregulated		[86]
Prostate cancer	miR-21, miR-99b/miR100, miR-24-2/miR-27a, miR-15a-2/ miR-195, miR-224, miR-10 (a/b), miR-125b (− 1/2), miR-30a/b/c-2, miR-26a (− 1/2), miR-126, miR-145, miR-151, miR-181a-1, let-7g	Upregulated	metallothionein	[45]
	miR-191	Upregulated	HRE2	[84]
	miR-124-3p	Downregulated	SARM1	[118]
HNSCC	miR-137	Downregulated		[117]
	let-7d	Upregulated		[135]
OSCC	miR-21	Upregulated	PTEN	[15]
	miR-370	Downregulated	IRS-1	[115]
	miR-7	Downregulated	RAF-1	[116]
	let-7a	Downregulated		[134]
Sinonasal SCC	miR-9-3p	Upregulated		[87]
Cutaneous SCC	miR-205	Upregulated		[88]

## Crosstalk between miRNAs and other PNI related molecules

### Neurotrophic factors

Neurotrophic factors, including nerve growth factor (NGF), glial cell line-derived neurotrophic factor (GDNF) and NT3 secreted from nerve were rich in the nerve-tumor microenvironment and acted as prominent promoters in PNI through respectively combining with their receptor, tyrosine kinase A receptor (TRKA), GDNF family receptors (GFR) and NT-3, expressed by tumor cells of pancreatic, breast and bile duct carcinomas [7]. Montalban et al. reported that NGF could provoke phosphorylation of AKT and MAPK in a miR-21-dependent manner, thus increasing VEGF levels [143]. Rocío et al. revealed that NGF could negatively regulate miR-23 to accelerate ovarian cancer progression [144]. Upregulation of miR-205, a pro-PNI miRNA induced by GDNF, was certificated to promote proliferation and migration in glioma cell [145]. In addition, miR-30a orchestrated by Wnt/β-catenin pathway could advance glioma cell invasion through repressing NCAM [146]. All the afore mentioned studies indicated that the crosstalk between neurotrophic factors and miRNAs might manipulate the tumor PNI process, while underlying mechanisms awaited further investigation in the future.

### Matrix metalloproteinases (MMPs)

MMPs contribute to PNI primarily via extracellular matrix (ECM) degradation and the activation of pro-invasion factors. Recent investigation showed that miR-124 overexpression hindered MMP-9 synthesis and impeded invasion of renal cell carcinoma [147]. MiR-141 downregulation could boost invasion and migration ability via MMP-2 and MMP-9 in bladder cancer [148]. In liver cancer, miR-26 could suppress growth of tumor cells through sensitizing PI3K/Akt and NF-κB/MMP-9/VEGF pathways [149]. MiR-21 promoted migratory and invasive potential of hepatocellular carcinoma by upregulating MMP-2 and MMP-9 expression [150]. MiR-15 reduced glioma cell invasion and angiogenesis via targeting MMP-3 [151]. MiR-200 family and let-7 could regulate the MMP-14 expression, which was pivotal for the cleavage of ECM components and the activation of proMMP-2 in pancreatic cancers [152]. However, whether the interplay between the miRNAs and MMPs could encourage the initiation and development of PNI in tumors was still unknown.

### Chemokine

Presently, attention has been focused on the association between chemokine signaling axes and miRNAs in malignant tumors. CXCL12 and its receptor CXCR4 is highly expressed in the perineural niche of neurotropic tumors, including pancreatic, prostate and breast cancer. Shiri et al. demonstrated that CXCR4 advanced neuroblastoma growth and therapeutic resistance via miR-15/16-mediated ERK and BCL2/Cyclin D1 pathways [153]. MiR-204 accelerated the migration and invasion of lung cancer via CXCR4 [154]. In colorectal cancer, CXCL12/CXCR4 mediated miR-125b to induce invasion and confer 5-fluorouracil resistance [155], or sequestered miR-133a to promote inflammation through the activation of RhoA signaling [156]. Inhibition of CXCR4 induced cell apoptosis of acute myeloid leukemia through upregulation of miR-15/miR-16, which targeted BCL-2, MCL-1 and cyclin-D1 [157]. In pancreatic cancer, CXCR4 negatively regulated let-7a to elevate HMGA2 expression, hence promoting cell proliferation, metastasis and chemosensitivity [158].

## Reciprocal interaction between PNI-related miRNAs and microenvironment in PNI

**Fig. 4:**
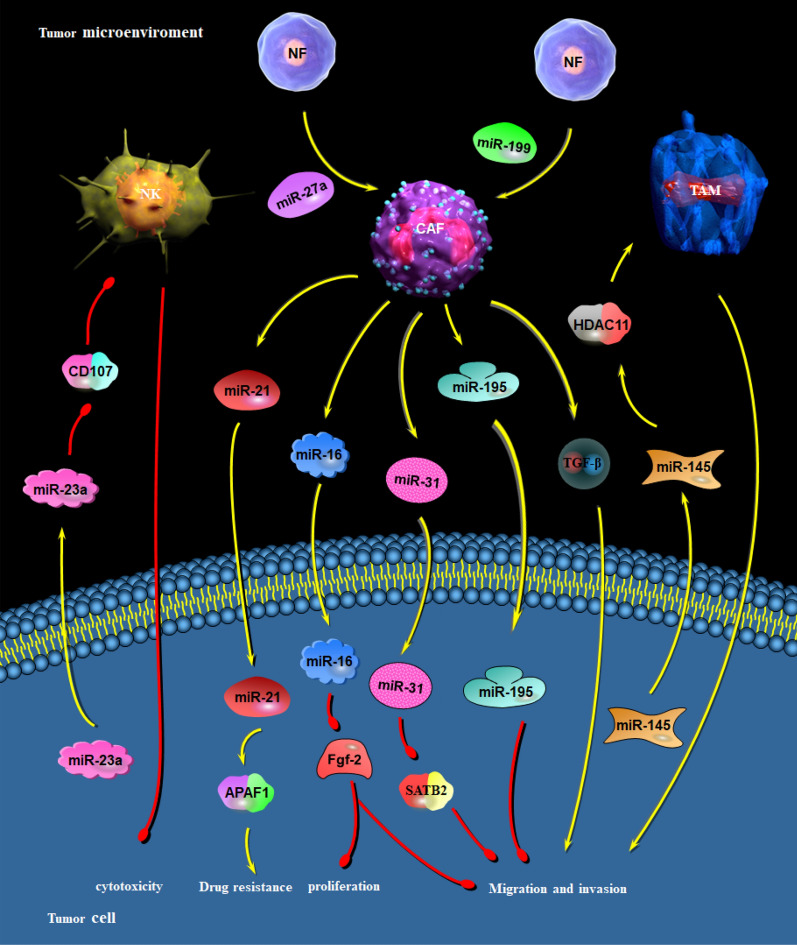
Reciprocal interaction between PNI related-miRNAs and stromal cells in tumor. MiR23a, generated by tumor cell in niche, inactivates NK cells through directly targeting CD107 expression and impairs their cytotoxicity against tumor cells. MiR-27a/b and miR-199 overexpression mediates a functional conversion of NFs into CAFs, which increases secretion of TGF-β and miR-21 to enhance migration, invasion and resistance of tumor cells to cisplatin, or upregulates miR-16, miR-31 and miR-195 to inhibit proliferation, migration and invasion of tumor cell. MiR-145, released by colorectal cancer cells, is transported to TAMs and educates the differentiation of TAMs towards M2-like phenotype, boosting migration and invasion of tumor cells

Copious research have disclosed the involvement of miRNAs in communication between tumor cells and stromal cells, including Treg cells, tumor-associated macrophages (TAMs) and cancer-associated fibroblasts (CAFs), which constitutes a bridge and is conducive to cancer cells influencing and hijacking the physiological processes, and establishing a favorable niche to engraft, expand and evade the immune surveillance and realizing PNI [159] (Fig. 4).

MiR23a could be conveyed from IGR-Heu (lung carcinoma cell line) and K562 (Leukemia cell line) to niche via microvesicles, thereby inactivating natural killer (NK) cells through directly targeting CD107 expression and impairing their cytotoxicity against tumor cells [160]. MiR-145, another pro-PNI miRNA, generated by colorectal cancer cells in the form of microvesicles, are transported to TAMs, where it weakens histone deacetylase 11 (HDAC11) expression, consequently educating and accelerating their differentiation towards M2-like phenotype and boosting tumor progression [161]. MiR-27a/b overexpression mediates a functional conversion of NFs into CAFs, which increases secretion of TGF-β, resulting in resistance of tumor cells to cisplatin in esophageal cancer [162]. MiR-199 upregulation could activate NFs and convert them into CAFs, enhancing proliferative and migratory capabilities of pancreatic cancer cell [163]. Cancer cell-released exosomal miR-21 enhances angiogenesis and neoplastic processes [164], in turn, miR-21 released by CAFs is responsible for resistance of ovarian cancer cells to paclitaxel by targeting APAF1 [165].

Conversely, miR-31, an anti-PNI miRNA, was downregulated distinctly in CAFs from endometrial cancer, and restoration of miR-31 could cripple cell migration and invasion by directly downregulating its target SATB2 [166]. CAF-derived exosomes could repress the growth, invasion and metastasis of tumor cell by carrying miR-195 in cholangiocarcinoma [167]. Reduction of miR-16 in CAFs facilitates proliferation and migration through reversing the post-transcriptional repression of Fgf-2 and its receptor Fgfr1 within the prostate cancer niche [168].

## Conclusion and prospective

PNI is a harbinger of occult nodal spread, providing a challenge to tumor eradication and portending a negative prognostic implication in malignancy of colon, pancreas and prostate. Recent findings have highlighted the essential roles of miRNAs, which can act not only as oncogene but as tumor suppressor in PNI, and discuss the potential biological mechanisms of miRNAs in the formation of pre-PNI niches and the molecular basis. However, we still lack a systematic framework of the whole process of cancer cells to encroach on nerve, and the specific principles of miRNAs controlling PNI are much more intricate, theoretically, these following unresolved issues seem worthy of further exploration:

### PNI and collective invasion

**Fig. 5:**
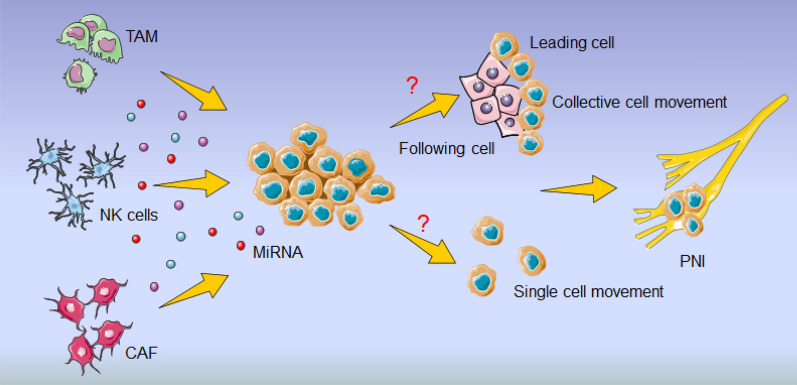
Prediction of PNI invasion pattern. MiRNAs constitutes a bridge of the crosstalk between tumor cells and stromal cells, including natural killer (NK) cells including regulatory T cells (Treg cells), tumor-associated macrophages (TAMs) and cancer-associated fibroblasts (CAFs), and regulates the process of PNI. How does miRNAs mediate PNI, through collective invasion, single cell invasion or both?

To encroach on nerve, tumor cells need to gain malignant phenotypes to detach from the primary tumor mass and evade the immune surveillance in neuro-tumor microenvironment. Collective cell invasion is a movement pattern of multiple cells that retain cell–cell connections and migrate coordinately, which is a fundamental property of many types of cancers [169]. Distinct from single-cell motility, this migrating cohesive groups comprise two distinct subpopulations, including leader cells (exploring the microenvironment at the invasive frontier, paving the way and orchestrating the movement of the whole clusters) and follower cells (promoting leader cells polarization and influencing leader cells behavior to assist leader cells to acquire and consolidate leadership), maintain a front-rear polarity and cooperate in a hierarchical manner to enhance persistent invasion [170]. How does miRNAs mediate PNI, through collective invasion, single cell invasion or both? (Fig. 5).

### Circulating miRNAs and PNI

MiRNAs can be obtained from plasma, blood, urine, saliva, and other body fluids besides tumor tissues. Although the dysregulated expression of miRNAs has been reported to be significantly associated with various kinds of human carcinoma and nerve remodeling, whether circulating miRNAs implicates in the facilitation of cancer PNI will be interesting to investigate.

### Animal models and PNI

Most of the present data on miRNAs regulating PNI are actually derived from in vitro cells. Animal models including orthotopic models and heterotopic models, which can recapitulate neural sprouting and neural tracking as it occurs in humans, assess the pain and dysfunction related to cancer invasion and permit the images of tumor spreading by magnetic resonance, to allow a comprehensive investigation of miRNAs in PNI and provide preclinical evidence.

### Therapeutic implications and PNI

MiRNA-based therapeutics including miRNA mimics and antimiRs have been conducted over the years, whereas, only a small number of miRNA therapeutics have so far moved into clinical development due to the potential for degradation of oligonucleotides by RNases and difficulty in identification of the best miRNA candidates for each disease type. It is worth mentioning that in vitro and in vivo studies have revealed that miRNAs such as miR-200, let-7 and miR-34 could sensitize cancer cells to chemotherapy, and mimics of above miRNAs could be rationally combined with chemotherapeutics [171]. However, the synergistic effect of miRNAs with chemotherapeutics on PNI prevention remains margin.

## Data Availability

Not applicable.

## Abbreviations

Ago: Argonaute
BTG2: B-cell translocation gene 2
CAFs: Cancer-associated fibroblasts
CLL: Chronic lymphocytic leukemia
CRC: Colorectal cancer
DGCR8: Double stranded RNA binding protein
DRG: Dorsal root ganglion
ECM: Extracellular matrix
EGF: Epidermal growth factor
Exp5: Export protein exportin 5
GDNF: Glial cell line-derived neurotrophic factor
GFR: GDNF family receptors
HCC: Hepatocellular carcinoma
HDAC11: Histone deacetylase 11
HIF-1: Hypoxia-inducible factor-1
IGF-1R: Insulin-like growth factor 1 receptor
MiRNA: MicroRNA
MMPs: Matrix metalloproteinases
NEDD4L: Neural precursor cell expressed developmentally downregulated 4-like
NGF: Nerve growth factor
NK cell: Natural killer cell
NT-3: Neurotrophin-3
OAC: Oesophageal adenocarcinoma
OncomiR: Oncogenic miRNA
OSCC: Oral squamous cell carcinoma
PDAC: Pancreatic ductal adenocarcinoma
PDCD4: Programmed cell death 4
PNI: Perineural invasion
Pol II: RNA polymerase II
Pre-miRNA: Precursor miRNA
Pri-miRNA: Primary miRNA precursor
PTEN: Phosphatase and tensin homolog deleted on chromosome ten
Ran-GTP: GTP-binding nuclear protein Ran
RISC: R NA-induced silencing complex
TAMs: Tumor-associated macrophages
TGF-β: Transforming growth factor-β
TIMP3: Tissue inhibitor of matrix metalloproteinases 3
TPM1: Tropomyosin-1
Treg cells: Regulatory T cells
TRKA: Tyrosine kinase A receptor
TRPM3: Transient receptor potential melastatin 3
3’-UTR: 3’-Untranslated region

## References

[1] Liebig C, Ayala G, Wilks JA, Berger DH, Albo D (2009). Perineural invasion in cancer: a review of the literature. Cancer.

[2] Lesnik DJ, Boey HP (2004). Perineural invasion of the facial nerve by a cutaneous squamous cell cancer: a case report. Ear Nose Throat J.

[3] Pour PM, Bell RH, Batra SK (2003). Neural invasion in the staging of pancreatic cancer. Pancreas.

[4] Huang T, Fan Q, Wang Y, Cui Y, Wang Z, Yang L, Sun X, Wang Y (2020). Schwann cell-derived CCL2 promotes the perineural invasion of cervical cancer. Front Oncol.

[5] Zhu S, Mendenhall WM (2018). Radiotherapy for melanoma with perineural invasion: university of florida experience. Cancer Invest.

[6] Amit M, Binenbaum Y, Trejo-Leider L, Sharma K, Ramer N, Ramer I, Agbetoba A, Miles B, Yang X, Lei D, Bjørndal K, Godballe C, Mücke T (2015). International collaborative validation of intraneural invasion as a prognostic marker in adenoid cystic carcinoma of the head and neck. Head Neck.

[7] Amit M, Na'ara S, Gil Z (2016). Mechanisms of cancer dissemination along nerves. Nat Rev Cancer.

[8] Marchesi F, Piemonti L, Fedele G, Destro A, Roncalli M, Albarello L, Doglioni C, Anselmo A, Doni A, Bianchi P, Laghi L, Malesci A, Cervo L (2008). The chemokine receptor CX3CR1 is involved in the neural tropism and malignant behavior of pancreatic ductal adenocarcinoma. Cancer Res.

[9] Batsakis JG (1985). Nerves and neurotropic carcinomas. Ann Otol Rhinol Laryngol.

[10] Liang D, Shi S, Xu J, Zhang B, Qin Y, Ji S, Xu W, Liu J, Liu L, Liu C, Long J, Ni Q, Yu X (2016). New insights into perineural invasion of pancreatic cancer: more than pain. Biochim Biophys Acta.

[11] Ceyhan GO, Demir IE, Altintas B, Rauch U, Thiel G, Müller MW, Giese NA, Friess H, Schäfer KH (2008). Neural invasion in pancreatic cancer: a mutual tropism between neurons and cancer cells. Biochem Biophys Res Commun.

[12] Jurcak N, Zheng L (2019). Signaling in the microenvironment of pancreatic cancer: transmitting along the nerve. Pharmacol Ther.

[13] Gasparini G, Pellegatta M, Crippa S, Lena MS, Belfiori G, Doglioni C, Taveggia C, Falconi M (2019). Nerves and pancreatic cancer: new insights into a dangerous relationship. Cancers Basel.

[14] Liu D, Song L, Dai Z, Guan H, Kang H, Zhang Y, Yan W, Zhao X, Zhang S (2018). MiR-429 suppresses neurotrophin-3 to alleviate perineural invasion of pancreatic cancer. Biochem Biophys Res Commun.

[15] Yu EH, Tu HF, Wu CH, Yang CC, Chang KW (2017). MicroRNA-21 promotes perineural invasion and impacts survival in patients with oral carcinoma. J Chin Med Assoc.

[16] Lewis BP, Shih IH, Jones-Rhoades MW, Bartel DP, Burge CB (2003). Prediction of mammalian microRNA targets. Cell.

[17] Salmena L, Poliseno L, Tay Y, Kats L, Pandolfi PP (2011). A ceRNA hypothesis: the Rosetta stone of a hidden RNA language?. Cell.

[18] Ha M, Kim VN (2014). Regulation of microRNA biogenesis. Nat Rev Mol Cell Biol.

[19] Lee Y, Kim M, Han J, Yeom KH, Lee S, Baek SH, Kim VN (2004). MicroRNA genes are transcribed by RNA polymerase II. Embo j.

[20] Lee Y, Ahn C, Han J, Choi H, Kim J, Yim J, Lee J, Provost P, Rådmark O, Kim S, Kim VN (2003). The nuclear RNase III Drosha initiates microRNA processing. Nature.

[21] Lund E, Güttinger S, Calado A, Dahlberg JE, Kutay U (2004). Nuclear export of microRNA precursors. Science.

[22] Xhemalce B, Robson SC, Kouzarides T (2012). Human RNA methyltransferase BCDIN3D regulates microRNA processing. Cell.

[23] Ruby JG, Jan CH, Bartel DP (2007). Intronic microRNA precursors that bypass Drosha processing. Nature.

[24] Okamura K, Hagen JW, Duan H, Tyler DM, Lai EC (2007). The mirtron pathway generates microRNA-class regulatory RNAs in Drosophila. Cell.

[25] Cheloufi S, Dos Santos CO, Chong MM, Hannon GJ (2010). A dicer-independent miRNA biogenesis pathway that requires Ago catalysis. Nature.

[26] Cifuentes D, Xue H, Taylor DW, Patnode H, Mishima Y, Cheloufi S, Ma E, Mane S, Hannon GJ, Lawson ND, Wolfe SA, Giraldez AJ (2010). A novel miRNA processing pathway independent of Dicer requires Argonaute2 catalytic activity. Science.

[27] Bartel DP (2009). MicroRNAs: target recognition and regulatory functions. Cell.

[28] Filipowicz W, Bhattacharyya SN, Sonenberg N (2008). Mechanisms of post-transcriptional regulation by microRNAs: are the answers in sight?. Nat Rev Genet.

[29] Hayes J, Peruzzi PP, Lawler S (2014). MicroRNAs in cancer: biomarkers, functions and therapy. Trends Mol Med.

[30] Sun HL, Cui R, Zhou J, Teng KY, Hsiao YH, Nakanishi K, Fassan M, Luo Z, Shi G, Tili E, Kutay H, Lovat F, Vicentini C (2016). ERK activation globally downregulates miRNAs through phosphorylating exportin-5. Cancer Cell.

[31] Henry JC, Azevedo-Pouly AC, Schmittgen TD (2011). MicroRNA replacement therapy for cancer. Pharm Res.

[32] Bertoli G, Cava C, Castiglioni I (2015). MicroRNAs: new biomarkers for diagnosis, prognosis, therapy prediction and therapeutic tools for breast cancer. Theranostics.

[33] Li M, Cui X, Guan H (2020). MicroRNAs: pivotal regulators in acute myeloid leukemia. Ann Hematol.

[34] Chan JA, Krichevsky AM, Kosik KS (2005). MicroRNA-21 is an antiapoptotic factor in human glioblastoma cells. Cancer Res.

[35] Strickland IT, Richards L, Holmes FE, Wynick D, Uney JB, Wong LF (2011). Axotomy-induced miR-21 promotes axon growth in adult dorsal root ganglion neurons. PLoS ONE.

[36] Buller B, Liu X, Wang X, Zhang RL, Zhang L, Hozeska-Solgot A, Chopp M, Zhang ZG (2010). MicroRNA-21 protects neurons from ischemic death. FEBS J.

[37] Sakai A, Suzuki H (2013). Nerve injury-induced upregulation of miR-21 in the primary sensory neurons contributes to neuropathic pain in rats. Biochem Biophys Res Commun.

[38] Hori N, Narita M, Yamashita A, Horiuchi H, Hamada Y, Kondo T, Watanabe M, Igarashi K, Kawata M, Shibasaki M, Yamazaki M, Kuzumaki N, Inada E (2016). Changes in the expression of IL-6-mediated MicroRNAs in the dorsal root ganglion under neuropathic pain in mice. Synapse.

[39] Lin L, Fan Y, Gao F, Jin L, Li D, Sun W, Li F, Qin P, Shi Q, Shi X, Du L (2018). UTMD-promoted co-delivery of gemcitabine and miR-21 inhibitor by dendrimer-entrapped gold nanoparticles for pancreatic cancer therapy. Theranostics.

[40] Itani S, Kunisada T, Morimoto Y, Yoshida A, Sasaki T, Ito S, Ouchida M, Sugihara S, Shimizu K, Ozaki T (2012). MicroRNA-21 correlates with tumorigenesis in malignant peripheral nerve sheath tumor (MPNST) via programmed cell death protein 4 (PDCD4). J Cancer Res Clin Oncol.

[41] Cioffi JA, Yue WY, Mendolia-Loffredo S, Hansen KR, Wackym PA, Hansen MR (2010). MicroRNA-21 overexpression contributes to vestibular schwannoma cell proliferation and survival. Otol Neurotol.

[42] Teplyuk NM, Mollenhauer B, Gabriely G, Giese A, Kim E, Smolsky M, Kim RY, Saria MG, Pastorino S, Kesari S, Krichevsky AM (2012). MicroRNAs in cerebrospinal fluid identify glioblastoma and metastatic brain cancers and reflect disease activity. Neuro Oncol.

[43] Singh M, Garg N, Venugopal C, Hallett R, Tokar T, McFarlane N, Mahendram S, Bakhshinyan D, Manoranjan B, Vora P, Qazi M, Arpin CC, Page B (2015). STAT3 pathway regulates lung-derived brain metastasis initiating cell capacity through miR-21 activation. Oncotarget.

[44] Guglielmi L, Nardella M, Musa C, Cifola I, Porru M, Cardinali B, Iannetti I, Di Pietro C, Bolasco G, Palmieri V, Vilardo L, Panini N, Bonaventura F (2020). Circulating miRNAs in small extracellular vesicles secreted by a human melanoma xenograft in mouse brains. Cancers (Basel).

[45] Prueitt RL, Yi M, Hudson RS, Wallace TA, Howe TM, Yfantis HG, Lee DH, Stephens RM, Liu CG, Calin GA, Croce CM, Ambs S (2008). Expression of microRNAs and protein-coding genes associated with perineural invasion in prostate cancer. Prostate.

[46] Frampton AE, Castellano L, Colombo T, Giovannetti E, Krell J, Jacob J, Pellegrino L, Roca-Alonso L, Funel N, Gall TM, Ahmad R, Habib NA, Knösel T (2015). Integrated molecular analysis to investigate the role of microRNAs in pancreatic tumour growth and progression. Lancet.

[47] Huang Q, Liu L, Liu CH, You H, Shao F, Xie F, Lin XS, Hu SY, Zhang CH (2013). MicroRNA-21 regulates the invasion and metastasis in cholangiocarcinoma and may be a potential biomarker for cancer prognosis. Asian Pac J Cancer Prev.

[48] Heimberg AM, Sempere LF, Moy VN, Donoghue PC, Peterson KJ (2008). MicroRNAs and the advent of vertebrate morphological complexity. Proc Natl Acad Sci U S A.

[49] Sun D, Lee YS, Malhotra A, Kim HK, Matecic M, Evans C, Jensen RV, Moskaluk CA, Dutta A (2011). miR-99 family of MicroRNAs suppresses the expression of prostate-specific antigen and prostate cancer cell proliferation. Cancer Res.

[50] Owen JL, Mohamadzadeh M (2013). Macrophages and chemokines as mediators of angiogenesis. Front Physiol.

[51] Cobos Jiménez V, Bradley EJ, Willemsen AM, van Kampen AH, Baas F, Kootstra NA (2014). Next-generation sequencing of microRNAs uncovers expression signatures in polarized macrophages. Physiol Genomics.

[52] Jaiswal A, Reddy SS, Maurya M, Maurya P, Barthwal MK (2019). MicroRNA-99a mimics inhibit M1 macrophage phenotype and adipose tissue inflammation by targeting TNFα. Cell Mol Immunol.

[53] Bouchareychas L, Duong P, Covarrubias S, Alsop E, Phu TA, Chung A, Gomes M, Wong D, Meechoovet B, Capili A, Yamamoto R, Nakauchi H, McManus MT (2020). Macrophage exosomes resolve atherosclerosis by regulating hematopoiesis and inflammation via MicroRNA Cargo. Cell Rep.

[54] Wang W, Liu Y, Guo J, He H, Mi X, Chen C, Xie J, Wang S, Wu P, Cao F, Bai L, Si Q, Xiang R (2018). miR-100 maintains phenotype of tumor-associated macrophages by targeting mTOR to promote tumor metastasis via Stat5a/IL-1ra pathway in mouse breast cancer. Oncogenesis.

[55] Cavel O, Shomron O, Shabtay A, Vital J, Trejo-Leider L, Weizman N, Krelin Y, Fong Y, Wong RJ, Amit M, Gil Z (2012). Endoneurial macrophages induce perineural invasion of pancreatic cancer cells by secretion of GDNF and activation of RET tyrosine kinase receptor. Cancer Res.

[56] Wang M, Gu H, Qian H, Zhu W, Zhao C, Zhang X, Tao Y, Zhang L, Xu W (2013). miR-17-5p/20a are important markers for gastric cancer and murine double minute 2 participates in their functional regulation. Eur J Cancer.

[57] Liu SQ, Jiang S, Li C, Zhang B, Li QJ (2014). miR-17-92 cluster targets phosphatase and tensin homology and Ikaros Family Zinc Finger 4 to promote TH17-mediated inflammation. J Biol Chem.

[58] Luan Y, Chen M, Zhou L (2017). MiR-17 targets PTEN and facilitates glial scar formation after spinal cord injuries via the PI3K/Akt/mTOR pathway. Brain Res Bull.

[59] Wang Z, Yuan W, Li B, Chen X, Zhang Y, Chen C, Yu M, Xiu Y, Li W, Cao J, Wang X, Tao W, Guo X (2019). PEITC promotes neurite growth in primary sensory neurons via the miR-17-5p/STAT3/GAP-43 axis. J Drug Target.

[60] Hasvik E, Schjølberg T, Jacobsen DP, Haugen AJ, Grøvle L, Schistad EI, Gjerstad J (2019). Up-regulation of circulating microRNA-17 is associated with lumbar radicular pain following disc herniation. Arthritis Res Ther.

[61] Wang Q, Zhan Y, Ren N, Wang Z, Zhang Q, Wu S, Li H (2018). Paraquat and MPTP alter microRNA expression profiles, and downregulated expression of miR-17-5p contributes to PQ-induced dopaminergic neurodegeneration. J Appl Toxicol.

[62] Li Z, Peng Z, Gu S, Zheng J, Feng D, Qin Q, He J (2017). Global analysis of miRNA-mRNA interaction network in breast cancer with brain metastasis. Anticancer Res.

[63] Gu J, Wang D, Zhang J, Zhu Y, Li Y, Chen H, Shi M, Wang X, Shen B, Deng X, Zhan Q, Wei G, Peng C (2016). GFRα2 prompts cell growth and chemoresistance through down-regulating tumor suppressor gene PTEN via Mir-17-5p in pancreatic cancer. Cancer Lett.

[64] Zhou Q, Gallagher R, Ufret-Vincenty R, Li X, Olson EN, Wang S (2011). Regulation of angiogenesis and choroidal neovascularization by members of microRNA-23~27~24 clusters. Proc Natl Acad Sci U S A.

[65] Chhabra R, Dubey R, Saini N (2010). Cooperative and individualistic functions of the microRNAs in the miR-23a~27a~24-2 cluster and its implication in human diseases. Mol Cancer.

[66] Yao C, Wang Y, Zhang H, Feng W, Wang Q, Shen D, Qian T, Liu F, Mao S, Gu X, Yu B (2018). lncRNA TNXA-PS1 modulates schwann cells by functioning as a competing endogenous RNA following nerve injury. J Neurosci.

[67] Cheng L, Wang C, Yao F, Li Z, Liu W, Jing J (2020). MicroRNA-26b inhibits oligodendrocyte precursor cell differentiation by targeting adrenomedullin in spinal cord injury. J Cell Physiol.

[68] Deng X, Liang C, Qian L, Zhang Q (2021). miR-24 targets HMOX1 to regulate inflammation and neurofunction in rats with cerebral vasospasm after subarachnoid hemorrhage. Am J Transl Res.

[69] Xu G, Li JY (2016). ATP5A1 and ATP5B are highly expressed in glioblastoma tumor cells and endothelial cells of microvascular proliferation. J Neurooncol.

[70] Chen L, Zhang A, Li Y, Zhang K, Han L, Du W, Yan W, Li R, Wang Y, Wang K, Pu P, Jiang T, Jiang C (2013). MiR-24 regulates the proliferation and invasion of glioma by ST7L via β-catenin/Tcf-4 signaling. Cancer Lett.

[71] Chen H, Lu Q, Chen C, Di Y, Li Y, Min W, Yu Z, Dai D (2019). β-catenin regulates effects of miR-24 on the viability and autophagy of glioma cells. Exp Ther Med.

[72] Croce CM (2009). Causes and consequences of microRNA dysregulation in cancer. Nat Rev Genet.

[73] Han J, Chen Q (2015). MiR-16 modulate temozolomide resistance by regulating BCL-2 in human glioma cells. Int J Clin Exp Pathol.

[74] Zhu D, Tu M, Zeng B, Cai L, Zheng W, Su Z, Yu Z (2017). Up-regulation of miR-497 confers resistance to temozolomide in human glioma cells by targeting mTOR/Bcl-2. Cancer Med.

[75] Yang TQ, Lu XJ, Wu TF, Ding DD, Zhao ZH, Chen GL, Xie XS, Li B, Wei YX, Guo LC, Zhang Y, Huang YL, Zhou YX (2014). MicroRNA-16 inhibits glioma cell growth and invasion through suppression of BCL2 and the nuclear factor-κB1/MMP9 signaling pathway. Cancer Sci.

[76] Krell A, Wolter M, Stojcheva N, Hertler C, Liesenberg F, Zapatka M, Weller M, Malzkorn B, Reifenberger G (2019). MiR-16-5p is frequently down-regulated in astrocytic gliomas and modulates glioma cell proliferation, apoptosis and response to cytotoxic therapy. Neuropathol Appl Neurobiol.

[77] Renjie W, Haiqian L (2015). MiR-132, miR-15a and miR-16 synergistically inhibit pituitary tumor cell proliferation, invasion and migration by targeting Sox5. Cancer Lett.

[78] Chen LP, Zhang NN, Ren XQ, He J, Li Y (2018). miR-103/miR-195/miR-15b Regulate SALL4 and Inhibit Proliferation and Migration in Glioma. Molecules..

[79] Wang X, Li XD, Fu Z, Zhou Y, Huang X, Jiang X (2020). Long non-coding RNA LINC00473/miR-195-5p promotes glioma progression via YAP1-TEAD1-Hippo signaling. Int J Oncol.

[80] Song LR, Li D, Weng JC, Li CB, Wang L, Wu Z, Zhang JT (2020). MicroRNA-195 functions as a tumor suppressor by directly targeting fatty acid synthase in malignant meningioma. World Neurosurg.

[81] Li T, Wan Y, Sun L, Tao S, Chen P, Liu C, Wang K, Zhou C, Zhao G (2019). Inhibition of MicroRNA-15a/16 expression alleviates neuropathic pain development through upregulation of G protein-coupled receptor kinase 2. Biomol Ther (Seoul).

[82] Wang X, Wang H, Zhang T, He M, Liang H, Wang H, Xu L, Chen S, Xu M (2019). Inhibition of MicroRNA-195 alleviates neuropathic pain by targeting patched1 and inhibiting SHH signaling pathway activation. Neurochem Res.

[83] Yin KJ, Deng Z, Huang H, Hamblin M, Xie C, Zhang J, Chen YE (2010). miR-497 regulates neuronal death in mouse brain after transient focal cerebral ischemia. Neurobiol Dis.

[84] Song Z, Ren H, Gao S, Zhao X, Zhang H, Hao J (2014). The clinical significance and regulation mechanism of hypoxia-inducible factor-1 and miR-191 expression in pancreatic cancer. Tumour Biol.

[85] Bai J, Zhang X, Shi D, Xiang Z, Wang S, Yang C, Liu Q, Huang S, Fang Y, Zhang W, Song J, Xiong B (2021). Exosomal miR-128–3p promotes epithelial-to-mesenchymal transition in colorectal cancer cells by targeting FOXO4 via TGF-β/SMAD and JAK/STAT3 signaling. Front Cell Dev Biol.

[86] Li M, Wang Q, Xue F, Wu Y (2019). lncRNA-CYTOR works as an oncogene through the CYTOR/miR-3679-5p/MACC1 axis in colorectal cancer. DNA Cell Biol.

[87] Kovaříková J, Baranová I, Laco J, Rozkošová K, Vošmíková H, Vošmík M, Dundr P, Němejcová K, Michálek J, Palička V, Chmelařová M (2019). Deregulation of selected MicroRNAs in sinonasal squamous cell carcinoma: searching for potential prognostic biomarkers. Folia Biol (Praha).

[88] Cañueto J, Cardeñoso-Álvarez E, García-Hernández JL, Galindo-Villardón P, Vicente-Galindo P, Vicente-Villardón JL, Alonso-López D, De Las RJ, Valero J, Moyano-Sanz E, Fernández-López E, Mao JH, Castellanos-Martín A (2017). MicroRNA (miR)-203 and miR-205 expression patterns identify subgroups of prognosis in cutaneous squamous cell carcinoma. Br J Dermatol.

[89] Tao X, Shao Y, Lu R, Ye Q, Xiao B, Ye G, Guo J (2020). Clinical significance of hsa_circ_0000419 in gastric cancer screening and prognosis estimation. Pathol Res Pract.

[90] Stepicheva NA, Song JL (2016). Function and regulation of microRNA-31 in development and disease. Mol Reprod Dev.

[91] Hua D, Ding D, Han X, Zhang W, Zhao N, Foltz G, Lan Q, Huang Q, Lin B (2012). Human miR-31 targets radixin and inhibits migration and invasion of glioma cells. Oncol Rep.

[92] Zhou RJ, Xu XY, Liu BX, Dai WZ, Cai MQ, Bai CF, Zhang XF, Wang LM, Lin L, Jia SZ, Wang WH (2015). Growth-inhibitory and chemosensitizing effects of microRNA-31 in human glioblastoma multiforme cells. Int J Mol Med.

[93] Li Z, Gu X, Fang Y, Xiang J, Chen Z (2012). microRNA expression profiles in human colorectal cancers with brain metastases. Oncol Lett.

[94] Chang HL, Wang HC, Chunag YT, Chou CW, Lin IL, Lai CS, Chang LL, Cheng KI (2017). miRNA expression change in dorsal root ganglia after peripheral nerve injury. J Mol Neurosci.

[95] Sarcognato S, Gringeri E, Fassan M, Di Giunta M, Maffeis V, Guzzardo V, Cillo U, Guido M (2019). Prognostic role of BAP-1 and PBRM-1 expression in intrahepatic cholangiocarcinoma. Virchows Arch.

[96] Wong TS, Liu XB, Wong BY, Ng RW, Yuen AP, Wei WI (2008). Mature miR-184 as potential oncogenic microRNA of squamous cell carcinoma of tongue. Clin Cancer Res.

[97] Szafranska AE, Davison TS, John J, Cannon T, Sipos B, Maghnouj A, Labourier E, Hahn SA (2007). MicroRNA expression alterations are linked to tumorigenesis and non-neoplastic processes in pancreatic ductal adenocarcinoma. Oncogene.

[98] Qin Y, Dang X, Li W, Ma Q (2013). miR-133a functions as a tumor suppressor and directly targets FSCN1 in pancreatic cancer. Oncol Res.

[99] Wei W, Liu Y, Lu Y, Yang B, Tang L (2017). LncRNA XIST promotes pancreatic cancer proliferation through miR-133a/EGFR. J Cell Biochem.

[100] Chang LL, Wang HC, Tseng KY, Su MP, Wang JY, Chuang YT, Wang YH, Cheng KI (2020). Upregulation of miR-133a-3p in the sciatic nerve contributes to neuropathic pain development. Mol Neurobiol.

[101] Raheja R, Regev K, Healy BC, Mazzola MA, Beynon V, Von Glehn F, Paul A, Diaz-Cruz C, Gholipour T, Glanz BI, Kivisakk P, Chitnis T, Weiner HL (2018). Correlating serum micrornas and clinical parameters in amyotrophic lateral sclerosis. Muscle Nerve.

[102] Rau CS, Jeng JC, Jeng SF, Lu TH, Chen YC, Liliang PC, Wu CJ, Lin CJ, Hsieh CH (2010). Entrapment neuropathy results in different microRNA expression patterns from denervation injury in rats. BMC Musculoskelet Disord.

[103] Lu XC, Zheng JY, Tang LJ, Huang BS, Li K, Tao Y, Yu W, Zhu RL, Li S, Li LX (2015). MiR-133b promotes neurite outgrowth by targeting RhoA expression. Cell Physiol Biochem.

[104] Xin H, Li Y, Liu Z, Wang X, Shang X, Cui Y, Zhang ZG, Chopp M (2013). MiR-133b promotes neural plasticity and functional recovery after treatment of stroke with multipotent mesenchymal stromal cells in rats via transfer of exosome-enriched extracellular particles. Stem Cells.

[105] Kim J, Inoue K, Ishii J, Vanti WB, Voronov SV, Murchison E, Hannon G, Abeliovich A (2007). A MicroRNA feedback circuit in midbrain dopamine neurons. Science.

[106] Dai Q, Ma Y, Xu Z, Zhang L, Yang H, Liu Q, Wang J (2021). Downregulation of circular RNA HECTD1 induces neuroprotection against ischemic stroke through the microRNA-133b/TRAF3 pathway. Life Sci.

[107] Shiels A (2020). TRPM3_miR-204: a complex locus for eye development and disease. Hum Genomics.

[108] Natera-Naranjo O, Aschrafi A, Gioio AE, Kaplan BB (2010). Identification and quantitative analyses of microRNAs located in the distal axons of sympathetic neurons. RNA.

[109] López-González MJ, Soula A, Landry M, Favereaux A (2018). Oxaliplatin treatment impairs extension of sensory neuron neurites in vitro through miR-204 overexpression. Neurotoxicology.

[110] Wang N, Yang W, Xiao T, Miao Z, Luo W, You Z, Li G (2018). Possible role of miR-204 in optic nerve injury through the regulation of GAP-43. Mol Med Rep.

[111] Gao R, Wang L, Sun J, Nie K, Jian H, Gao L, Liao X, Zhang H, Huang J, Gan S (2014). MiR-204 promotes apoptosis in oxidative stress-induced rat Schwann cells by suppressing neuritin expression. FEBS Lett.

[112] Mohammed CP, Rhee H, Phee BK, Kim K, Kim HJ, Lee H, Park JH, Jung JH, Kim JY, Kim HC, Park SK, Nam HG, Kim K (2016). miR-204 downregulates EphB2 in aging mouse hippocampal neurons. Aging Cell.

[113] Gong M, Ma J, Li M, Zhou M, Hock JM, Yu X (2012). MicroRNA-204 critically regulates carcinogenesis in malignant peripheral nerve sheath tumors. Neuro Oncol.

[114] Tan X, Huang Z, Li X (2017). Long non-coding RNA MALAT1 interacts with miR-204 to modulate human hilar cholangiocarcinoma proliferation, migration, and invasion by targeting CXCR4. J Cell Biochem.

[115] Chang KW, Chu TH, Gong NR, Chiang WF, Yang CC, Liu CJ, Wu CH, Lin SC (2013). miR-370 modulates insulin receptor substrate-1 expression and inhibits the tumor phenotypes of oral carcinoma. Oral Dis.

[116] Dou Z, Gao L, Ren W, Zhang H, Wang X, Li S, Zheng J, Kong X, Chi P, Zhi K (2020). CiRS-7 functions as a ceRNA of RAF-1/PIK3CD to promote metastatic progression of oral squamous cell carcinoma via MAPK/AKT signaling pathways. Exp Cell Res.

[117] Čelešnik H, Büdefeld T, Čizmarević B, Švagan M, Potočnik U (2021). MIR137/MIR2682 locus is associated with perineural invasiveness in head and neck cancer. J Oral Pathol Med.

[118] Yan K, Hou L, Liu T, Jiao W, Ma Q, Fang Z, Zhang S, Song D, Liu J, Gao X, Fan Y (2020). lncRNA OGFRP1 functions as a ceRNA to promote the progression of prostate cancer by regulating SARM1 level via miR-124-3p. Aging (Albany NY).

[119] Xie L, Zhang Z, Tan Z, He R, Zeng X, Xie Y, Li S, Tang G, Tang H, He X (2014). MicroRNA-124 inhibits proliferation and induces apoptosis by directly repressing EZH2 in gastric cancer. Mol Cell Biochem.

[120] Li L, Luo J, Wang B, Wang D, Xie X, Yuan L, Guo J, Xi S, Gao J, Lin X, Kong Y, Xu X, Tang H (2013). Microrna-124 targets flotillin-1 to regulate proliferation and migration in breast cancer. Mol Cancer.

[121] Zheng F, Liao YJ, Cai MY, Liu YH, Liu TH, Chen SP, Bian XW, Guan XY, Lin MC, Zeng YX, Kung HF, Xie D (2012). The putative tumour suppressor microRNA-124 modulates hepatocellular carcinoma cell aggressiveness by repressing ROCK2 and EZH2. Gut.

[122] Lu J, Li X, Wang F, Guo Y, Huang Y, Zhu H (2017). YB-1 expression promotes pancreatic cancer metastasis that is inhibited by microRNA-216a. Exp Cell Res.

[123] Pandey A, Singh P, Jauhari A, Singh T, Khan F, Pant AB, Parmar D, Yadav S (2015). Critical role of the miR-200 family in regulating differentiation and proliferation of neurons. J Neurochem.

[124] Choi PS, Zakhary L, Choi WY, Caron S, Alvarez-Saavedra E, Miska EA, McManus M, Harfe B, Giraldez AJ, Horvitz HR, Schier AF, Dulac C (2008). Members of the miRNA-200 family regulate olfactory neurogenesis. Neuron.

[125] Yang C, Zhang X, Yin H, Du Z, Yang Z (2020). MiR-429/200a/200b negatively regulate Notch1 signaling pathway to suppress CoCl(2)-induced apoptosis in PC12 cells. Toxicol In Vitro.

[126] Minn YK, Lee DH, Hyung WJ, Kim JE, Choi J, Yang SH, Song H, Lim BJ, Kim SH (2014). MicroRNA-200 family members and ZEB2 are associated with brain metastasis in gastric adenocarcinoma. Int J Oncol.

[127] Reinhart BJ, Slack FJ, Basson M, Pasquinelli AE, Bettinger JC, Rougvie AE, Horvitz HR, Ruvkun G (2000). The 21-nucleotide let-7 RNA regulates developmental timing in *Caenorhabditis elegans*. Nature.

[128] Slack FJ, Basson M, Liu Z, Ambros V, Horvitz HR, Ruvkun G (2000). The lin-41 RBCC gene acts in the *C. elegans* heterochronic pathway between the let-7 regulatory RNA and the LIN-29 transcription factor. Mol Cell.

[129] Li S, Wang X, Gu Y, Chen C, Wang Y, Liu J, Hu W, Yu B, Wang Y, Ding F, Liu Y, Gu X (2015). Let-7 microRNAs regenerate peripheral nerve regeneration by targeting nerve growth factor. Mol Ther.

[130] Zhang J, Zhang Y, Chen L, Rao Z, Sun Y (2020). Ulinastatin promotes regeneration of peripheral nerves after sciatic nerve injury by targeting let-7 microRNAs and enhancing NGF expression. Drug Des Devel Ther.

[131] Wang X, Chen Q, Yi S, Liu Q, Zhang R, Wang P, Qian T, Li S (2019). The microRNAs let-7 and miR-9 down-regulate the axon-guidance genes Ntn1 and Dcc during peripheral nerve regeneration. J Biol Chem.

[132] Lehmann SM, Krüger C, Park B, Derkow K, Rosenberger K, Baumgart J, Trimbuch T, Eom G, Hinz M, Kaul D, Habbel P, Kälin R, Franzoni E (2012). An unconventional role for miRNA: let-7 activates Toll-like receptor 7 and causes neurodegeneration. Nat Neurosci.

[133] Fernández V, Martínez-Martínez M, Prieto-Colomina A, Cárdenas A, Soler R, Dori M, Tomasello U, Nomura Y, López-Atalaya JP, Calegari F, Borrell V (2020). Repression of Irs2 by let-7 miRNAs is essential for homeostasis of the telencephalic neuroepithelium. EMBO J.

[134] Brito BL, Lourenço SV, Damascena AS, Kowalski LP, Soares FA, Coutinho-Camillo CM (2016). Expression of stem cell-regulating miRNAs in oral cavity and oropharynx squamous cell carcinoma. J Oral Pathol Med.

[135] Kolenda T, Guglas K, Teresiak A, Bliźniak R, Lamperska K (2019). Low let-7d and high miR-205 expression levels positively influence HNSCC patient outcome. J Biomed Sci.

[136] Bao N, Fang B, Lv H, Jiang Y, Chen F, Wang Z, Ma H (2018). Upregulation of miR-199a-5p protects spinal cord against ischemia/reperfusion-induced injury via downregulation of ECE1 in rat. Cell Mol Neurobiol.

[137] Gao Z, Zhao Y, He X, Leng Z, Zhou X, Song H, Wang R, Gao Z, Wang Y, Liu J, Niu B, Li H, Ouyang P (2020). Transplantation of sh-miR-199a-5p-modified olfactory ensheathing cells promotes the functional recovery in rats with contusive spinal cord injury. Cell Transplant.

[138] Wang D, Li Z, Zhang Y, Wang G, Wei M, Hu Y, Ma S, Jiang Y, Che N, Wang X, Yao J, Yin J (2016). Targeting of microRNA-199a-5p protects against pilocarpine-induced status epilepticus and seizure damage via SIRT1-p53 cascade. Epilepsia.

[139] Liu G, Detloff MR, Miller KN, Santi L, Houlé JD (2012). Exercise modulates microRNAs that affect the PTEN/mTOR pathway in rats after spinal cord injury. Exp Neurol.

[140] Xu WH, Yao XY, Yu HJ, Huang JW, Cui LY (2012). Downregulation of miR-199a may play a role in 3-nitropropionic acid induced ischemic tolerance in rat brain. Brain Res.

[141] Lv HR (2017). lncRNA-Map2k4 sequesters miR-199a to promote FGF1 expression and spinal cord neuron growth. Biochem Biophys Res Commun.

[142] Sousa LO, Sobral LM, Matsumoto CS, Saggioro FP, López RV, Panepucci RA, Curti C, Silva WA, Greene LJ, Leopoldino AM (2016). Lymph node or perineural invasion is associated with low miR-15a, miR-34c and miR-199b levels in head and neck squamous cell carcinoma. BBA Clin.

[143] Montalban E, Mattugini N, Ciarapica R, Provenzano C, Savino M, Scagnoli F, Prosperini G, Carissimi C, Fulci V, Matrone C, Calissano P, Nasi S (2014). MiR-21 is an Ngf-modulated microRNA that supports Ngf signaling and regulates neuronal degeneration in PC12 cells. Neuromol Med.

[144] Retamales-Ortega R, Oróstica L, Vera C, Cuevas P, Hernández A, Hurtado I, Vega M, Romero C (2017). Role of nerve growth factor (NGF) and miRNAs in epithelial ovarian cancer. Int J Mol Sci.

[145] Zhang BL, Dong FL, Guo TW, Gu XH, Huang LY, Gao DS (2017). MiRNAs mediate GDNF-induced proliferation and migration of glioma cells. Cell Physiol Biochem.

[146] Wang Z, Dai X, Chen Y, Sun C, Zhu Q, Zhao H, Liu G, Huang Q, Lan Q (2015). MiR-30a-5p is induced by Wnt/β-catenin pathway and promotes glioma cell invasion by repressing NCAM. Biochem Biophys Res Commun.

[147] Wang P, Zhang LD, Sun MC, Gu WD, Geng HZ (2018). Over-expression of mir-124 inhibits MMP-9 expression and decreases invasion of renal cell carcinoma cells. Eur Rev Med Pharmacol Sci.

[148] Liu W, Qi L, Lv H, Zu X, Chen M, Wang J, Liu L, Zeng F, Li Y (2015). MiRNA-141 and miRNA-200b are closely related to invasive ability and considered as decision-making biomarkers for the extent of PLND during cystectomy. BMC Cancer.

[149] Feng Y, Zu LL, Zhang L (2018). MicroRNA-26b inhibits the tumor growth of human liver cancer through the PI3K/Akt and NF-κB/MMP-9/VEGF pathways. Oncol Rep.

[150] Zhu Q, Wang Z, Hu Y, Li J, Li X, Zhou L, Huang Y (2012). miR-21 promotes migration and invasion by the miR-21-PDCD4-AP-1 feedback loop in human hepatocellular carcinoma. Oncol Rep.

[151] Zheng X, Chopp M, Lu Y, Buller B, Jiang F (2013). MiR-15b and miR-152 reduce glioma cell invasion and angiogenesis via NRP-2 and MMP-3. Cancer Lett.

[152] Ali S, Banerjee S, Logna F, Bao B, Philip PA, Korc M, Sarkar FH (2012). Inactivation of Ink4a/Arf leads to deregulated expression of miRNAs in K-Ras transgenic mouse model of pancreatic cancer. J Cell Physiol.

[153] Klein S, Abraham M, Bulvik B, Dery E, Weiss ID, Barashi N, Abramovitch R, Wald H, Harel Y, Olam D, Weiss L, Beider K, Eizenberg O (2018). CXCR4 promotes neuroblastoma growth and therapeutic resistance through miR-15a/16-1-mediated ERK and BCL2/Cyclin D1 pathways. Cancer Res.

[154] Liang T, Wang B, Li J, Liu Y (2019). LINC00922 Accelerates the proliferation, migration and invasion of lung cancer via the miRNA-204/CXCR4 axis. Med Sci Monit.

[155] Yu X, Shi W, Zhang Y, Wang X, Sun S, Song Z, Liu M, Zeng Q, Cui S, Qu X (2017). CXCL12/CXCR4 axis induced miR-125b promotes invasion and confers 5-fluorouracil resistance through enhancing autophagy in colorectal cancer. Sci Rep.

[156] Yu X, Wang D, Wang X, Sun S, Zhang Y, Wang S, Miao R, Xu X, Qu X (2019). CXCL12/CXCR4 promotes inflammation-driven colorectal cancer progression through activation of RhoA signaling by sponging miR-133a-3p. J Exp Clin Cancer Res.

[157] Abraham M, Klein S, Bulvik B, Wald H, Weiss ID, Olam D, Weiss L, Beider K, Eizenberg O, Wald O, Galun E, Avigdor A, Benjamini O (2017). The CXCR4 inhibitor BL-8040 induces the apoptosis of AML blasts by downregulating ERK, BCL-2, MCL-1 and cyclin-D1 via altered miR-15a/16-1 expression. Leukemia.

[158] Xiao G, Wang X, Yu Y (2017). CXCR4/Let-7a axis regulates metastasis and chemoresistance of pancreatic cancer cells through targeting HMGA2. Cell Physiol Biochem.

[159] Yang F, Ning Z, Ma L, Liu W, Shao C, Shu Y, Shen H (2017). Exosomal miRNAs and miRNA dysregulation in cancer-associated fibroblasts. Mol Cancer.

[160] Berchem G, Noman MZ, Bosseler M, Paggetti J, Baconnais S, Le Cam E, Nanbakhsh A, Moussay E, Mami-Chouaib F, Janji B, Chouaib S (2016). Hypoxic tumor-derived microvesicles negatively regulate NK cell function by a mechanism involving TGF-β and miR23a transfer. Oncoimmunology.

[161] Shinohara H, Kuranaga Y, Kumazaki M, Sugito N, Yoshikawa Y, Takai T, Taniguchi K, Ito Y, Akao Y (2017). Regulated polarization of tumor-associated macrophages by miR-145 via colorectal cancer-derived extracellular vesicles. J Immunol.

[162] Tanaka K, Miyata H, Sugimura K, Fukuda S, Kanemura T, Yamashita K, Miyazaki Y, Takahashi T, Kurokawa Y, Yamasaki M, Wada H, Nakajima K, Takiguchi S (2015). miR-27 is associated with chemoresistance in esophageal cancer through transformation of normal fibroblasts to cancer-associated fibroblasts. Carcinogenesis.

[163] Kuninty PR, Bojmar L, Tjomsland V, Larsson M, Storm G, Östman A, Sandström P, Prakash J (2016). MicroRNA-199a and -214 as potential therapeutic targets in pancreatic stellate cells in pancreatic tumor. Oncotarget.

[164] Liu Y, Luo F, Wang B, Li H, Xu Y, Liu X, Shi L, Lu X, Xu W, Lu L, Qin Y, Xiang Q, Liu Q (2016). STAT3-regulated exosomal miR-21 promotes angiogenesis and is involved in neoplastic processes of transformed human bronchial epithelial cells. Cancer Lett.

[165] Au Yeung CL, Co NN, Tsuruga T, Yeung TL, Kwan SY, Leung CS, Li Y, Lu ES, Kwan K, Wong KK, Schmandt R, Lu KH, Mok SC (2016). Exosomal transfer of stroma-derived miR21 confers paclitaxel resistance in ovarian cancer cells through targeting APAF1. Nat Commun.

[166] Aprelikova O, Yu X, Palla J, Wei BR, John S, Yi M, Stephens R, Simpson RM, Risinger JI, Jazaeri A, Niederhuber J (2010). The role of miR-31 and its target gene SATB2 in cancer-associated fibroblasts. Cell Cycle.

[167] Li L, Piontek K, Ishida M, Fausther M, Dranoff JA, Fu R, Mezey E, Gould SJ, Fordjour FK, Meltzer SJ, Sirica AE, Selaru FM (2017). Extracellular vesicles carry microRNA-195 to intrahepatic cholangiocarcinoma and improve survival in a rat model. Hepatology.

[168] Musumeci M, Coppola V, Addario A, Patrizii M, Maugeri-Saccà M, Memeo L, Colarossi C, Francescangeli F, Biffoni M, Collura D, Giacobbe A, D'Urso L, Falchi M (2011). Control of tumor and microenvironment cross-talk by miR-15a and miR-16 in prostate cancer. Oncogene.

[169] Friedl P, Locker J, Sahai E, Segall JE (2012). Classifying collective cancer cell invasion. Nat Cell Biol.

[170] Chen BJ, Wu JS, Tang YJ, Tang YL, Liang XH (2020). What makes leader cells arise: intrinsic properties and support from neighboring cells. J Cell Physiol.

[171] Rupaimoole R, Slack FJ (2017). MicroRNA therapeutics: towards a new era for the management of cancer and other diseases. Nat Rev Drug Discov.

